# One-step primary endoscopic ultrasound-guided choledochoduodenostomy without lumen-apposing metal stent using a Franseen needle and an ultra-stiff high-sliding guidewire

**DOI:** 10.1055/a-2374-8711

**Published:** 2024-08-08

**Authors:** Tadahisa Inoue, Rena Kitano, Mayu Ibusuki, Tomoya Kitada, Kazumasa Sakamoto, Jun Arai, Kiyoaki Ito

**Affiliations:** 1 12703 Department of Gastroenterology, Aichi Medical University, Nagakute, Japan

Endoscopic ultrasound-guided choledochoduodenostomy (EUS-CDS) using a lumen-apposing metal stent (LAMS) can be used as a primary treatment for malignant distal biliary obstruction because of its higher technical success rates and shorter procedure times than conventional transpapillary metal stent placement 1 2 . However, it is unsuitable for minimally dilated common bile ducts (CBDs) and thin 6-mm-diameter LAMSs are commonly used owing to large flanges. Other disadvantages include high costs, early stent dysfunction, and adverse events caused by biliary wall compression, duodenobiliary reflux, and cautery puncture 3 . Conversely, EUS-CDS with conventional metal stents requires a fistula dilation step that is time-consuming, leading to biliary peritonitis and a high risk of stent migration. Therefore, we propose a novel one-step EUS-CDS method without a LAMS using a Franseen needle and an ultra-stiff, high-sliding guidewire.

The Franseen needle creates a larger-diameter fistula during puncture than standard needles 4 ( Fig. 1 ). The 0.035-inch guidewire has a thick, high-rigidity nickel-titanium core and polytetrafluoroethylene coating with “ridge-processing” to minimize the contact area and friction, enhancing device followability and insertability ( Fig. 2 ). This combination can eliminate the need for fistula dilation even when inserting a thick delivery system. A dumbbell-shaped metal stent 5 was employed for stenting owing to its antimigration properties.

**Fig. 1:**
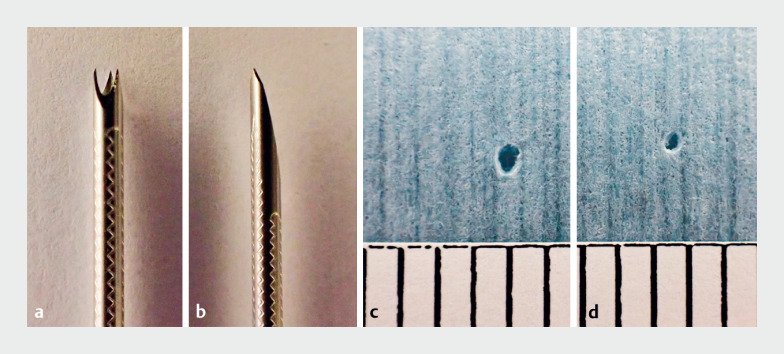
**a, c** In the bench test, the 19-G Franseen needle (SonoTip TopGain; Medi-Globe, Rohrdorf, Germany) creates a 1.27 mm-diameter hole **. b, d** In contrast, the 19-G standard needle (SonoTip Pro Control; Medi-Globe) creates a 0.70 mm-diameter hole.

**Fig. 2:**
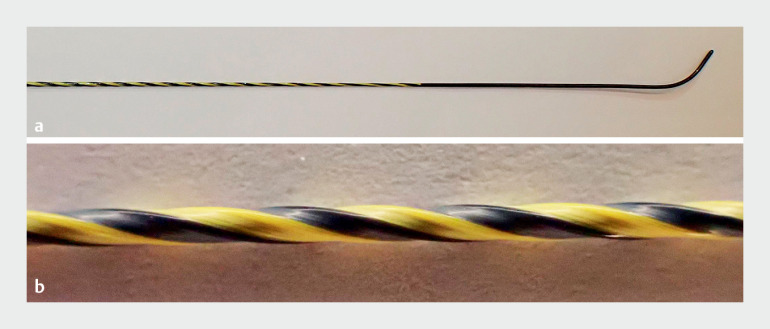
The novel guidewire measures 0.035 inches and features a thick, high-rigidity nickel-titanium core. The surface is coated with polytetrafluoroethylene, using “ridge-processing”, which reduces contact area and friction with devices, enhancing followability and insertability.

An 83-year-old man with obstructive jaundice due to malignant distal biliary obstruction was scheduled for primary EUS-CDS drainage. After CBD puncture from the duodenum using a 19-G Franseen needle (SonoTip TopGain; Medi-Globe, Rohrdorf, Germany), an ultra-stiff guidewire (SeekMaster Hard; Piolax Medical Devices, Kanagawa, Japan) was inserted into the intrahepatic bile duct. Subsequently, the 8-Fr delivery system of the dumbbell-shaped stent (BONASTENT M-Intraductal; Standard Sci-Tech Inc., Seoul, Korea) was smoothly inserted without fistula dilation, followed by placement of the stent from the CBD to the duodenum ( Fig. 3 , Video 1 ). The procedure was completed within five minutes. No adverse events or stent dysfunction, including biliary peritonitis or migration, occurred until the patient’s death.

**Fig. 3:**
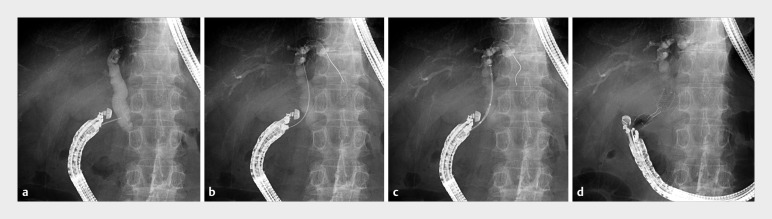
**a** A 19-G Franseen needle was used to puncture the common bile duct from the duodenum. **b** The novel guidewire was inserted into the intrahepatic bile duct. **c** Subsequently, the 8-Fr delivery system of the dumbbell-shaped metal stent was smoothly inserted without the need for fistula dilation. **d** The metal stent (12 × 50 mm) was then placed from the common bile duct to the duodenum.

One-step primary endoscopic ultrasound-guided choledochoduodenostomy using a Franseen needle and an ultra-stiff, high-sliding guidewireVideo 1

This method offers a straightforward and effective primary drainage approach for malignant distal biliary obstruction, addressing the limitations of EUS-CDS with a LAMS.

Endoscopy_UCTN_Code_TTT_1AS_2AH

## Notes

Endoscopy E-Videos https://eref.thieme.de/e-videos
*E-Videos* is an open access online section of the journal *Endoscopy* , reporting on interesting cases and new techniques in gastroenterological endoscopy. All papers include a high-quality video and are published with a Creative Commons CC-BY license. Endoscopy E-Videos qualify for HINARI discounts and waivers and eligibility is automatically checked during the submission process. We grant 100% waivers to articles whose corresponding authors are based in Group A countries and 50% waivers to those who are based in Group B countries as classified by Research4Life (see: https://www.research4life.org/access/eligibility/ ). This section has its own submission website at https://mc.manuscriptcentral.com/e-videos .
